# Examination of Homing Behaviors in Two Species of Crayfish Following Translational Displacements

**DOI:** 10.1093/iob/obz008

**Published:** 2019-04-20

**Authors:** Maryam Kamran, Meghan E Moore, Andrea M Fisher, Paul A Moore

**Affiliations:** 1 Department of Fisheries and Wildlife, Oregon State University, Corvallis, OR 97330, USA; 2 Department of Psychology, Bowling Green State University, Bowling Green, OH 43403, USA; 3 Department of Biological Sciences, University of South Florida St. Petersburg, St. Petersburg, FL 33701, USA; 4 J.P. Scott Center for Neuroscience, Mind & Behavior, Departments of Biological Sciences and Psychology, Bowling Green State University, Bowling Green, OH 43403, USA; 5 Laboratory for Sensory Ecology, Department of Biological Sciences, Bowling Green State University, Bowling Green, OH 43403, USA

## Abstract

Crayfish have been model systems for examining complex behaviors and the underlying neural mechanisms that guide these behaviors. While spatial learning has been examined in a subset of crayfish species, homing behaviors remained largely unexamined. Here we examined homing behavior following translational displacements in a primary burrowing (*Creaserinus fodiens*) and tertiary burrowing species (*Faxonius rusticus*). Individuals of both species were placed in an arena with artificial burrows embedded within the arena floor. The arena floor was fitted with a panel, which served as a treadmill belt to allow for translational displacement. Individuals were displaced after they had left the burrows. The movement pathways of displaced crayfish were compared with those in two control groups, one which underwent no displacement and the second in which the treadmill belt was displaced but returned to its original position almost immediately. Homing success for displaced individuals of both species was considerably reduced in comparison to the control groups. Moreover, displaced primary burrowers had significantly lower homing success in comparison to displaced tertiary burrowers. Primary burrowers exhibited greater homing error and significantly impaired homing behaviors compared with tertiary burrowers. Furthermore, heading angles in displaced groups (of both species) were significantly higher than the control group of both species. Species-specific differences in homing success and homing error indicate that primary burrowers were more negatively impacted by translational displacements. These homing differences indicate that these two species of crayfish have differing homing strategies.

## Synopsis

Examen del Instinto de Regresar al Hogar en Dos Especies de Cangrejos de Río Después de Desplazamientos Translacionales (Examination of Homing Behaviors in Two Species of Crayfish Following Translational Displacements) Los cangrejos de río han sido sistemas modelo para examinar comportamientos complejos y los mecanismos neuronales subyacentes que guían estos comportamientos. Si bien el aprendizaje espacial se ha examinado en un subconjunto de especies de cangrejos de río, los comportamientos de regresar al hogar permanecieron en gran parte sin examinar. Aquí se examinó el comportamiento de regresar al hogar luego de los desplazamientos traslacionales en una especie de madriguera primaria (*Creaserinus fodiens*) y de madriguera terciaria (*Faxonius rusticus*). Los individuos de ambas especies se colocaron en una arena con madrigueras artificiales incrustadas dentro del piso de la arena. El piso de arena estaba equipado con un panel, que servía como cinta de correr para permitir el desplazamiento traslacional. Los individuos fueron desplazados después de haber abandonado las madrigueras. Las vías de movimiento de los cangrejos de río desplazados se compararon con las de dos grupos de control, uno que no experimentó ningún desplazamiento y el segundo en el que se desplazó el cinturón de la cinta de corer pero fue devuelto a su posición original casi de inmediato. El éxito de regresar a casa para los individuos desplazados de ambas especies se redujo considerablemente en comparación con los grupos de control. Además, los excavadores primarios desplazados tuvieron significativamente menos éxito de regresar al hogar en comparación con los excavadores terciarios desplazados. Los excavadores primarios exhibieron un mayor error de regresar al hogar y el desempeño de ese comportamiento también fue significativamente deteriorado en comparación con los excavadores terciarios. Además, los ángulos de encabezamiento en los grupos desplazados (de ambas especies) fueron significativamente más altos que el grupo de control de ambas especies. Las diferencias específicas de la especie en el éxito de regresar al hogar y el error de la orientación indican que las excavaciones primarias se vieron afectadas más negativamente por los desplazamientos traslacionales. Estas diferencias de orientación indican que estas dos especies de cangrejos de río tienen diferentes estrategias de localización. translated to Spanish by Y.E. Jimenez (yordano_jimenez@brown.edu)

Examinando Comportamentos de Entocamento em Duas Espécies de Lagostins Após Deslocamentos Translationais (Examination of Homing Behaviors in Two Species of Crayfish Following Translational Displacements) Os lagostins têm sido usados como sistemas-modelo para examinar comportamentos complexos e os mecanismos neurais subjacentes que ditam esses comportamentos. Embora a aprendizagem espacial tenha sido examinada em um subconjunto de espécies de lagostins, os comportamentos de entocamento permaneceram em grande parte sem estudo. Aqui, foram examinados os comportamentos de encotamento após deslocamentos translacionais em espécies fossoriais primárias (*Creaserinus fodiens*) e terciárias (*Faxonius rusticus*). Indivíduos de ambas as espécies foram colocados em uma arena com tocas artificiais embutidas no chão da arena. O piso foi equipado com um painel que serviu como uma esteira para permitir o deslocamento translacional. Indivíduos foram deslocados depois de terem deixado as tocas. As rotas de movimentação dos lagostins deslocados foram comparadas com as de dois grupos-controle: um que não sofreu deslocamento e um segundo cuja esteira foi deslocada, mas retornou à sua posição original quase imediatamente. O sucesso no alojamento de indivíduos deslocados de ambas as espécies foi consideravelmente reduzido em comparação com os grupos-controle. Além disso, os indivíduos fossoriais primários deslocados tiveram um sucesso significativamente menor em comparação com os fossoriais terciários deslocados. Fossoriais primários exibiram maior erro de entocamento e tiveram os comportamentos de entocamento significativamente prejudicados em comparação aos fossoriais terciários. Ademais, os ângulos de direção nos grupos deslocados (de ambas as espécies) foram significativamente maiores que os do grupo-controle de ambas as espécies. Diferenças específicas para cada espécie no sucesso e no erro de entocamento indicam que as espécies fossoriais primárias foram mais impactadas negativamente pelos deslocamentos translacionais. Essas diferenças indicam que as duas espécies de lagostins têm diferentes estratégias de orientação. translated to Portuguese by G. Sobral (gabisobral@gmail.com)

Untersuchung des Heimfindeverhaltens in Zwei Flusskrebsarten Nach translatorischer Verschiebung (Examination of Homing Behaviors in Two Species of Crayfish Following Translational Displacements) Flusskrebse wurden als Modellsysteme für die Untersuchung komplexer Verhaltensweisen und der zugrunde liegenden neuronalen Mechanismen verwendet. Während das räumliche Lernen in einem Teil der Flusskrebsarten untersucht wurde, blieb das Heimfindeverhalten weitgehend ununtersucht. Hier untersuchten wir das Heimfindeverhalten nach translatorischer Verschiebung in einer primär (*Creaserinus fodiens*) und einer tertiär grabenden Flusskrebsart (*Faxonius rusticus*). Individuen beider Arten wurden in eine Arena mit im Boden eingelassenen künstlichen Höhlen platziert. Der Boden der Arena war mit einer Platte ausgestattet, die als Laufband diente, um eine translatorische Verschiebung vorzunehmen. Die Individuen wurden versetzt, nachdem sie die Höhlen verlassen hatten. Die Bewegungsbahnen der versetzten Flusskrebse wurden mit denen in zwei Kontrollgruppen verglichen, eine, die keine Versetzung erfuhr, und die zweite, in der das Laufband bewegt wurde, aber fast sofort in seine ursprüngliche Position zurückkehrte. Der Heimkehrerfolg für versetzte Individuen beider Arten war im Vergleich zu den Kontrollgruppen erheblich reduziert. Außerdem hatten versetzte primär grabende Flusskrebse einen signifikant niedrigeren Heimkehrerfolg als tertiär grabende. Primär grabende Flusskrebse zeigten im Vergleich mit tertiär grabenden einen größeren Fehler beim Heimkehren und ein signifikant beeinträchtigtes Heimkehrverhalten. Darüber hinaus waren die Bewegungswinkel in versetzten Gruppen (beider Arten) signifikant höher als die der Kontrollgruppen beider Arten. Artspezifische Unterschiede im Erfolg des Heimkehrens und Fehler im Heimkehren weisen darauf hin, dass primär grabende Flusskrebse durch translatorische Verschiebung stärker negativ beeinflusst werden. Diese Unterschiede im Heimkehren weisen darauf hin, dass diese beiden Flusskrebsarten unterschiedliche Heimkehr-Strategien haben. translated to German by F. Klimm (frederike.klimm@neptun.uni-freiburg.de)

## Introduction

An essential tool for the survival of organisms is their ability to navigate between resource rich areas. Broadly defined, the movement of organisms between resource areas can be defined as movement toward a goal (Hansson and Åkesson 2014). As a subset of these movement patterns, homing can be defined as the movement of an animal returning to a known location, often a nest, burrow, or some form of shelter (Able 1980; Hansson and Åkesson 2014). These goal directed homing behaviors can be differentiated by the information utilized to find a remote homing goal. This sensory information used to guide movement patterns is frequently divided into two categories, allothetic information or idiothetic information (Schöne 1984). Allothetic information relies on sensory cues external to the animal. As such, allothetic strategies for homing use directional information that is spatially associated with the homing location but independent from the animal’s movement in space. Allothetic information encompasses a range of homing cues from compass cues, such as the use of the earth’s magnetic field (Lohmann et al. 1995; Boles and Lohmann 2003; Ernst and Lohmann 2016), to the use of visual cues such as visual panoramas and landmarks (Tinbergen and Kruyt 1938; Kim et al. 2010; Warrant and Dacke 2010; Jin et al. 2014).

In contrast, idiothetic homing uses internal cues as sources of information (Mittelstaedt and Mittelstaedt 1973; Jander 1975). Path integration is a prime example of the use of idiothetic cues to home. Organisms that home using path integration continuously calculate a homing vector as they move throughout their spatial environment and thus follow a direct route to their homing goal (Muller and Wehner 1988; Collett and Collett 2000; Wehner and Srinivasan 2003). These homing vectors are calculated internally by determining both the distance and direction traveled from the home location (Seelig and Jayaraman 2015; Collett and Collett 2017). As an organism moves throughout its environment, this vector is recalculated using the previous distance and heading angle calculations in addition to any new movement. Research in arthropods has demonstrated that these animals rely on internally stored information based on the animals’ own movements, more specifically the use of proprioceptors on walking legs to determine distance (Seyfarth and Barth 1972; Mittelstaedt and Mittelstaedt 1973; Jander 1975; Seyfarth et al. 1982; Wittlinger et al. 2006). The importance of walking legs for the accurate calculation of home vectors is further emphasized in fiddler crabs which were found to miscalculate the distance to a burrow after manipulations, such as slipping on an acetate sheet, which induced an error in their walking paths (Layne et al. 2003a). Many arthropods combine information on movement extracted from visual cues along with idiothetic information to perform homing behaviors (Collett and Collett 2017). Differences in idiothetic homing mechanisms exist across species and across habitats.

To further understand the complex underlying mechanisms of homing behaviors, we tested homing behavior in two sympatric species of crayfish. These two species of crayfish create burrows of differing complexity. Primary burrowers, such as *Creaserinus fodiens* (the digger crayfish) build deep, complex burrows, and often spend a significant portion of their lives within the burrow (Atkinson and Eastman 2015). In contrast, tertiary burrowers build simple, shallow burrows which are often small single channel burrows or modified substrate. Tertiary burrowers spend considerable amounts of time outside of the burrow structure and may use abandoned burrows, built by other crayfish, as their own. A prime example of tertiary burrowers are the invasive crayfish, *Faxonius rusticus* (rusty crayfish). Consequently, primary burrowers invest more energy and effort into their burrows and the selective pressure to home to their burrow may be higher than tertiary burrowers who invest less energy into their burrows. This difference of investment in burrow use and construction between species creates a possible difference in the homing mechanisms.

The purpose of this study was to determine the homing mechanism used by primary and tertiary burrowing crayfish. To test which homing strategy animals are using, we intentionally induce an error in their walking paths by displacing the crayfish. If path integration is the primary mechanism of homing, we would predict that crayfish would exhibit a decrease in homing success and end their homing path at a distance equivalent to that of the displacement. For example, if the crayfish was displaced 10 cm away from a burrow, their path would end 10 cm before the burrow entrance. Altering the distance between the crayfish and the burrow, in this manner allowed us to determine if idiothetic information is used. Conversely, if the displaced crayfish were to successfully return to their burrow after displacement, there exists the possibility of the use of allothetic cues to supplement idiothetic cues.

## Materials and methods

### Animals

Two species of crayfish were used in this experiment, *F.rusticus* and *C.fodiens*. The *F.rusticus* were collected from branches of the Portage River, Wood County, OH, USA (41.361398°, −83.591038°) while *C.fodiens* were collected from a local unnamed pond (41.355585°, −83.862049°), in Wood County, OH, USA. Crayfish were sized matched as closely as possible with *F. rusticus* averaging a carapace length of 2.87 ± 0.4 cm and *C. fodiens* averaging 2.14 ± 0.12 cm (carapace) in length. All animals were housed in individual flow-through plastic containers to ensure visual and mechanical isolation. Animals were kept in a climate controlled chamber with a 12 h:12 h light:dark cycle and a constant temperature of 22.5 ± 0.5°C. All individuals were fed three times weekly with commercial-size rabbit food pellets (Forti-Diet, Central Garden and Pet, Walnut Creek, CA, USA). Animals were deprived of food for 10 days prior to the start of the experiment to increase motivation for foraging. Individuals were used only once during the trials.

### Experimental arena

**Fig. 1:**
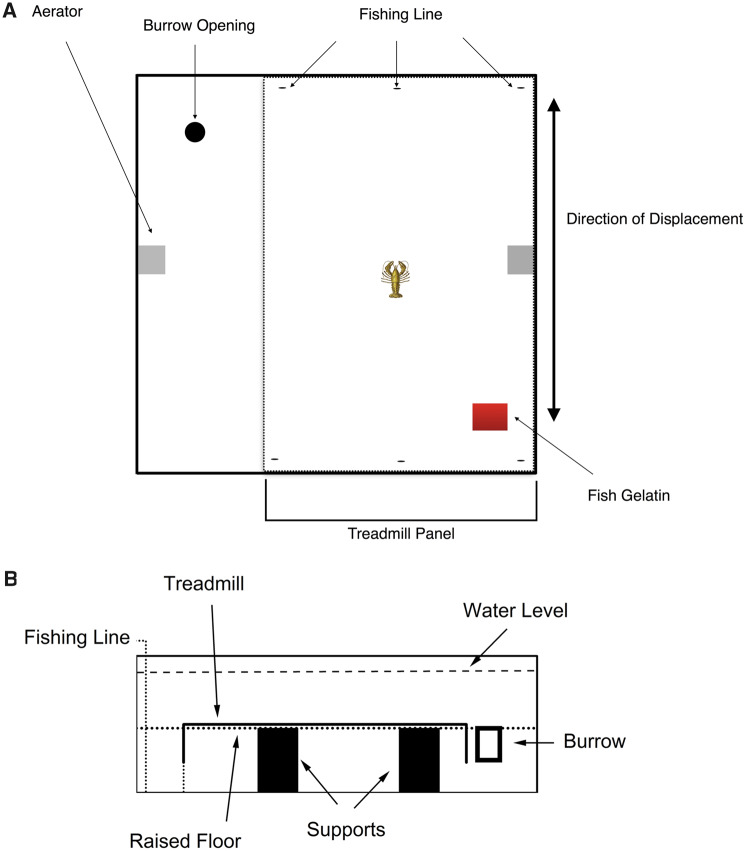
A) A top down view of the experimental arena highlighting the treadmill portion of the arena floor. The displacement of the treadmill allowed for a linear movement, either forward or backward using the fishing lines. **B**) Schematic of the arena and treadmill showing the elevated floor and fishing line to pull the treadmill.

Trials were conducted in one of two identical homing arenas, each arena measuring 80 cm × 80 cm × 35.5 cm (L × W × H). Both arenas were placed in an environmental chamber in which temperature was maintained at 23 ± 0.9°C and kept on a constant 12 h:12 h light:dark cycle. An artificial floor made from egg grating (1.3 cm holes) and mesh wire (0.13 cm holes) was elevated 17 cm above the base of each arena and supported by PVC pipes. The artificial floor, sides of the arena, and walls of the environmental chamber were either white or painted white to enhance contrast against the crayfish and to eliminate possible visual cues. Previous trials have shown that the paint is not toxic to crayfish and does not alter their behaviors (Kamran et al. 2018). The arena floor was constructed using two panels of egg crating (54.6 × 74.9 cm and 21.6 × 74.9 cm). The smaller panel contained the burrow (described below) and the second panel was the treadmill (Fig. 1A). The treadmill belt (114.5 cm × 55 cm) was constructed using a second sheet of mesh wiring with nickel washers attached at either end of the mesh with silicone. Preliminary trials showed that the additional weight provided by the washers attached to the mesh allowed for a smoother displacement. Attached to one end of the treadmill were three fishing lines (0.2 mm diameter) threaded through a series of eyelets that lead from the floor of the arena, up the sidewall, and out of the arena. The fishing lines allowed for smooth and consistent movement of the treadmill when pulled. The fishing lines allowed for a linear displacement of the treadmill belt in two directions—forward and backward. Additionally, a black cloth was placed around the arena to limit visual cues from the surrounding area. The arena was filled with artificial pond water and aerated prior to the start of each experiment. At the end of each trial, the arena was completely drained and refilled for the next experiment (Fig. 1B).

### Burrow

Previous work has shown that crayfish will readily use a PVC pipe as a burrow when provided (Kamran et al. 2018). An artificial burrow, constructed from 10.1 cm section of a 2.54 cm diameter PVC piped, was embedded in the raised floor of each arena and fully submerged in the water. The opening of the burrow was painted white and positioned at the same height as the raised floor. Finally, the inside walls and base of the PVC burrow were coated with a layer of sand to provide traction for the animals.

### Food stimulus

Fish gelatin was used in each trial to provide motivation for the crayfish to leave and return to the burrows during the trial (Willman et al. 1994; Wolf et al. 2004). The gelatin was made using 46 g of homogenized canned sardines in oil blended with 600 mL boiling water and four packets of Knox’s unflavored gelatin. The gelatin was poured into a ceramic pan, cooled in a refrigerator until hardened, and cut into usable sizes (2 cm × 2 cm × 1 cm).

### Behavioral assay protocol

At the start of each trial, a single crayfish was placed at the center of the arena. Crayfish were allowed a period of 4 h to find the burrow and explore the arena. Crayfish were allowed 2 h to find the burrow initially and an additional 2 h to exit and return to the burrow. Preliminary analysis indicated that this time period maximized the number of trials to collect useable data. Longer time periods did not significantly increase the number of crayfish that performed the required homing task. After the crayfish found the burrow, a piece of fish gelatin, in a weighted mesh bag, was placed near the burrow entrance and slowly moved to the opposite end of the arena. The mesh bag was left in the arena for the entire trial period. Once the crayfish exited the burrow and located the fish gelatin, one of three treatments were performed. The control treatment had an experimenter, behind the black sheeting, but the treadmill remained stationary. During a second control treatment, deemed the displaced-control treatment, an experimenter would displace the treadmill at set distance (11.5 ± 1.0 cm) and then return the treadmill to the original location. The third treatment was the displaced group, where an experiment would displace the treadmill and the treadmill would not be returned to the original location. There were two instances of tail flips in the displaced group and three in the displaced control. These individuals were dropped from subsequent analyses (*n* = 5). A total of 120 crayfish were used and only used once. The summary and *N*s of the treatments are:

**Table:** 

*F. rusticus*	
Control	20
Displaced-control	20
Displaced	20
*C. fodiens*	
Control	20
Displaced-control	20
Displaced	20

### Data analysis

All trials were video recorded with an overhead camera (SW PRO 530-4PK) using a SWANN DVR (model SWDVK-430004). The trials were recorded at one frame per second until the crayfish returned to the burrow or until the 2-h period following the fish gelatin placement expired. This temporal resolution was used as it has been used previously to describe the spatial parameters of movement pathways in crayfishes (Moore and Grills 1999; Kamran and Moore 2015; Moore et al. 2015; Kamran et al. 2018). A marker was placed at a single location on the carapace of a crayfish and used to track the *x*, *y* position of the crayfish throughout the trial. Videos of the recorded trials were digitized (one frame rate per second) using EthoVision XT 8.5 (Noldus Information Technology, The Netherlands) and Tracker (Open Source Physics, OSP, USA). The tracks were digitized at a single point per second using the center of the carapace as a reference point (Kamran and Moore 2015; Kamran et al. 2018). For a trial to be considered a successful trial, the crayfish must have exited a burrow and on the return journey must have reached either the burrow or the location at which the fictive burrow would have been. If the crayfish did not move for 3 s and began a localized search, the homing phase of the trial was considered to be done. Preliminary trial demonstrated that if 3 s passed without movement, animals were likely to incite a secondary search pattern rather than continue moving linearly, indicating that animals were no longer using path integration. Preliminary trials indicated that this time period was indicative of a change between homing and searching because the pause was followed by a local search pattern rather than a linear movement. For purpose of analysis, the outbound path, homing path, and locations of the burrow prior and post displacement were digitized. During digitizing, the track started when once the entire body of the crayfish had completely exited the burrow. Finally, digitized elements of the homing pathways were further analyzed by using previously defined homing parameters (Kamran and Moore 2015; Kamran et al. 2018). The heading angle on the animals home bound path to burrow opening was further analyzed. Heading angle was defined as the angle between the line from the animals current position (*t* = 0) to the burrow and the line from the animals current position (*t* = 0) to the animals next position (*t* = +1). An angle of 0° indicated the animal is pointing and moving toward the burrow. The distance to the burrow as well as the initial location of the burrow prior to displacement and post-displacement in all three groups (control, displaced-control, and displaced) were recorded. The distance between the location of the burrow and the end of a homing path is defined as the induced error. The return journey was defined as having begun when a crayfish had 10 consecutive points where the distance to the burrow decreased. This definition was used as a benchmark across trials to provide a starting point for the return pathway. Only the return pathways were used for homing behavior analysis as these pathways would most effectively indicate differences in homing behaviors between species and across treatment groups.

### Statistics

To discern differences between the two species as well as differences among treatments in regards to homing success, data were analyzed using a modified Chi-square analysis followed by a Tukey multiple proportions contingency table (Zar 2007). Because two different homing arenas were used, a non-linear mixed model in R (Bates et al. 2015; R Core Team 2018) was used for non-binary data such as heading angles and induced error. The behavioral variables model was initially constructed with full interactions using two factors (species and treatment) as well as a single random factor (homing tank). When significant differences were found with the interaction terms, differential contrasts were used with a Tukey-HSD *posthoc* test to determine where significant differences existed (Hothorn et al. 2008; R Core Team 2018). The Rayleigh test of significance of the mean angle was utilized when analyzing heading angles relative to the burrow (Agostinelli and Lund 2017). A Watson–Williams test for homogeneity of the means was conducted for the circular data (mean heading angles relative to burrow) in R (Agostinelli and Lund 2017).

## Results

### Displaced-controls

**Fig. 2:**
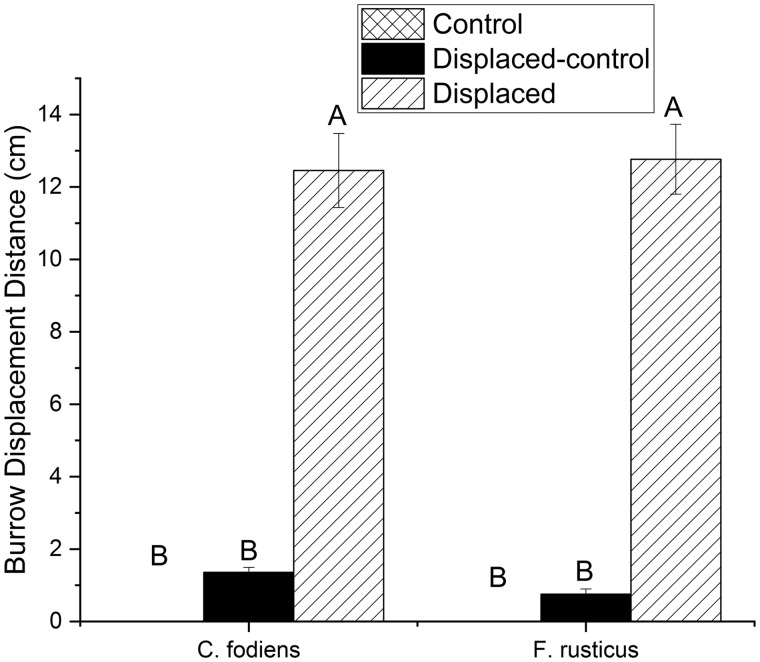
The mean (±SEM) displacement distance for control (cross hatched bar), displaced-control (solid black bar), and displaced (slanted bar) trials. *C. fodiens* are located on the left side and *F. rusticus* are on the right. *N* = 20 for each bar. Capital letters represent significant differences using a mixed model followed by a Tukey-HSD *post hoc* test (*P* < 0.05).

**Fig. 3:**
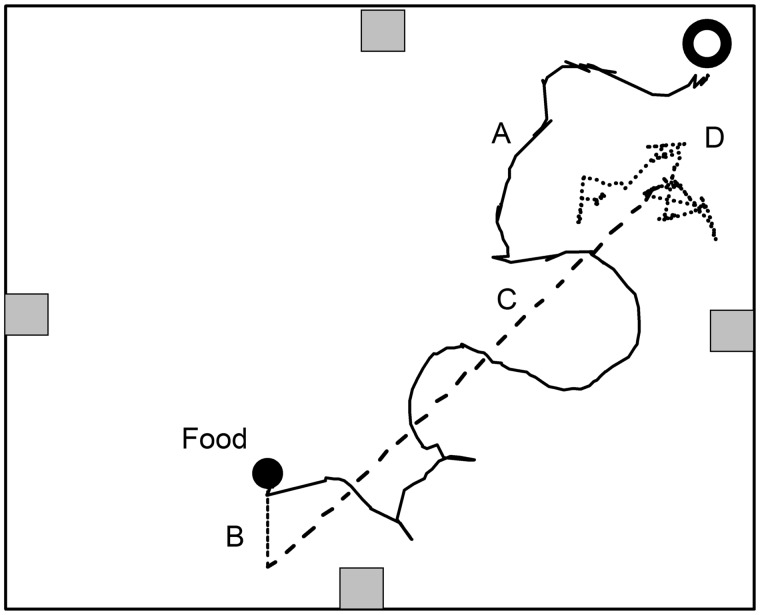
Top-down view of an arena (80 cm × 80 cm × 35.5 cm) with an example crayfish path digitized at 1 point per second. The gray squares along the sides represent aerators. The open black circle is the PVC burrow and the closed black circle is the location of the food resource. The crayfish path consists of the outbound search (**A**), the treadmill translation (**B**), the linear return path (**C**), and finally, a localized search pattern when the burrow is not found (**D**). This is a single example of a real homing pathway for a crayfish.

Displaced-control treatments had an average final error of 0.8 ± 0.14 cm from their original location. The average displacement for this group of trials was 1.27 ± 0.9 cm (Fig. 2). The controls treatments involved no movement. There was no significant difference between the final distance moved in the control and the displaced-control treatments. Figure 3 demonstrates a homing path for *F. rusticus* using the four elements of the homing strategy and the translation that occurred during the displacement (Fig. 3).

### Homing success

**Fig. 4:**
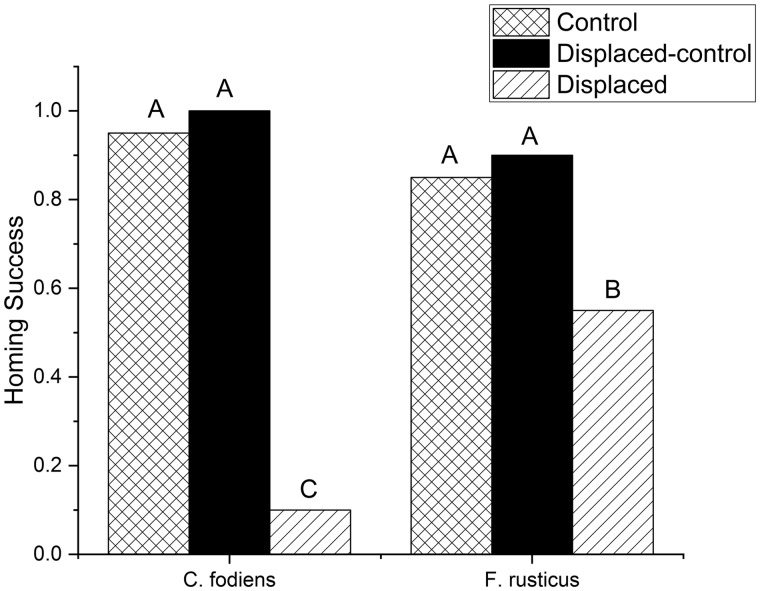
Homing success as a proportion of trials for control (cross hatched bar), displaced-control (solid black bar), and displaced (slanted bar) trials. *C. fodiens* are located on the left side and *F. rusticus* are on the right. *N* = 20 for each bar. Capital letters represent significant differences using a Tukey multiple proportions test (*P* < 0.05).

There was no significant difference in the ability of either crayfish species to home under either control homing condition (Tukey multiple proportions test, *P* > 0.8 for all comparisons: Fig. 4). *Creaserinusfodiens* successfully returned to the burrow 95% and 100% of the time for control and displaced-control trials, respectively. Similarly, *F. rusticus* also returned to the burrow in these two conditions, 85% for controls and 90% for displaced-controls. When the treadmill was displaced, both species of crayfish had significant deficits in their homing ability. *Creaserinusfodiens* returned to the burrow only 10% of the time and *F. rusticus* returned to the burrow 55% of the time (Fig. 4). Both treadmill displacement treatments were significantly different from a controls and from each other (Tukey multiple proportions test, *P* < 0.05).

### Overall homing parameters

The model indicated an overall significant interaction effect of species and treatment on the induced error (*F*_(2,143,0.05)_ = 4.34, *P* = 0.015). The heading angle only displayed a significant effect due to treatment (*F*_(2,143,0.05)_ = 25.2, *P* < 0.001), but no interaction or species effect (*P* = 0.9 and 0.9). Finally, the statistics on walking speed only found a significant difference across species (*F*_(2,143,0.05)_  = 12.1, *P* < 0.001), but not for treatment or the interaction between treatment and species (*P* = 0.89 and 0.13, respectively; Table 1).

**Table 1:** The mean values of the homing parameters measured for the return pathways of both species for each treatment are shown.

Homing parameter	Treatment	*F. rusticus*	*C. fodiens*
Walking speed (cm/s)	Control	1.3 ± 0.09	1.66 ± 0.13
	Displaced-control	1.3 ± 0.07	1.76 ± 0.1
	Displaced	1.43 ± 0.12	1.5 ± 0.12
Turning angle (degrees)	Control	44 ± 0.7	43 ± 0.6
	Displaced-control	45 ± 0.9	45 ± 0.9
	Displaced	44 ± 0.65	45 ± 0.9
Heading angle (degrees)	Control	2.3 ± 1.6	2.4 ± 1.6
	Displaced-control	358 ± 1.6	0.4 ± 1.3
	Displaced	49 ± 6.65	48 ± 6.0
Homing journey (s)	Control	27.9 ± 1.9	30 ± 2.2
	Displaced-control	26.3 ± 0.85	25.7 ± 0.89
	Displaced	27.8 ± 2.2	28.7 ± 2.9

### Induced error

**Fig. 5:**
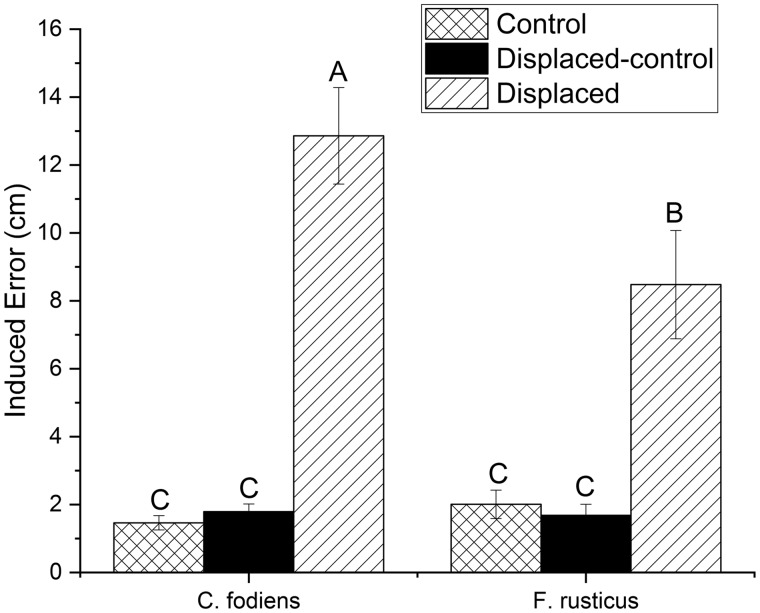
The mean (±SEM) induced error distance for control (cross hatched bar), displaced-control (solid black bar), and displaced (slanted bar) trials. *C. fodiens* are located on the left side and *F. rusticus* are on the right. *N* = 20 for each bar. Capital letters represent significant differences using a mixed model followed by a Tukey-HSD *post hoc* test (*P* < 0.05).

When displaced, both *C. fodiens* and *F. rusticus* failed to return to the burrow and underestimated the distance to the burrow by the displaced distance (Fig. 5). *Creaserinusfodiens*, in the displaced treatment, had an average induced error of 12.9 ± 1.4 cm which was significantly higher than the induced error of *F. rusticus* in the displaced treatment, 8.5 ± 1.6 cm (*P* < 0.001). Both of these groups had higher induced errors than either species in either of the controls (*C. fodiens*, control = 1.4 ± 0.2 cm, displaced-control = 1.8 ± 0.8 cm, *F. rusticus*, control = 2.0 ± 0.4 cm, and displaced-control = 1.7 ± 0.3 cm; *P* < 0.001).

### Heading angles

**Fig. 6:**
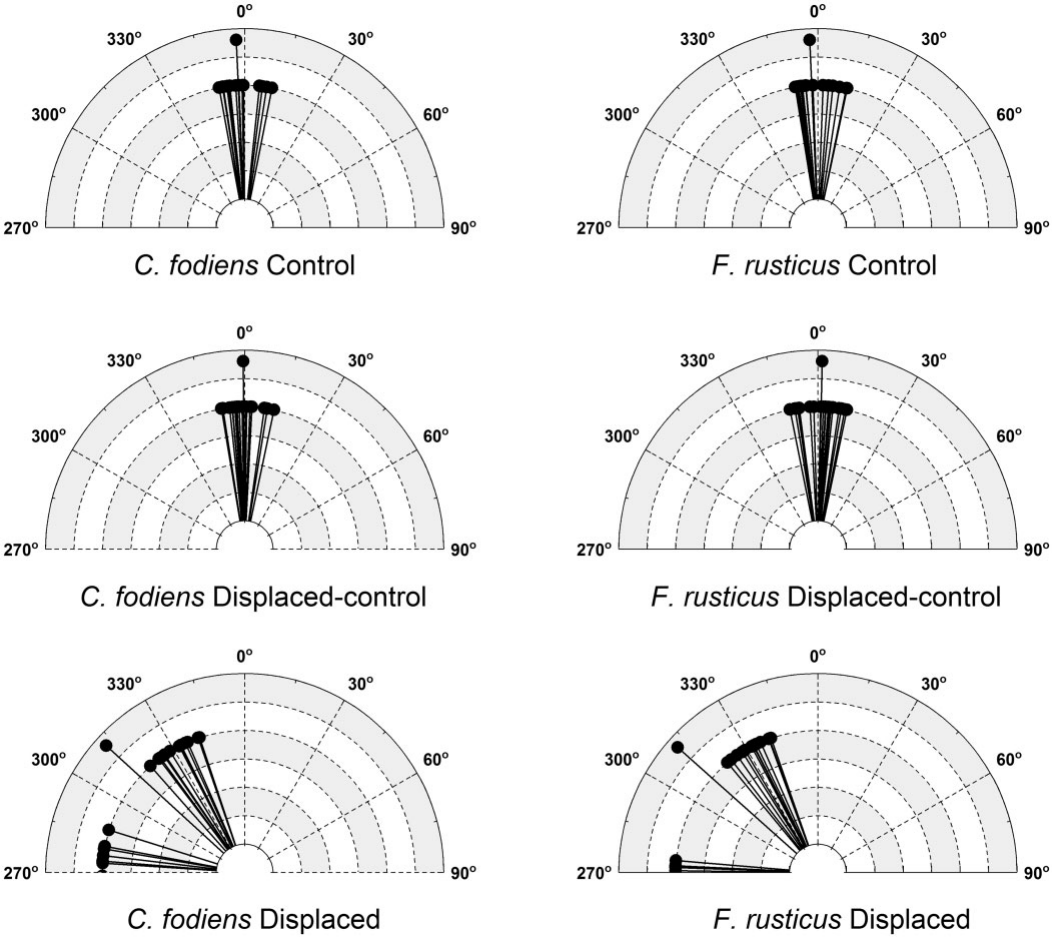
A cluster distribution of the mean heading angles on the return path for crayfish in the control (top graphs), displaced-control (middle graphs), and displaced (bottom graphs) trials. *C. fodiens* are located on the left side and *F. rusticus* are on the right. Mean angles are represented by the longer lines. Heading angles are calculated using the burrow regardless of displacement distance.

Both *C. fodiens* and *F. rusticus* exhibited significantly different heading angles relative to the burrow in the displaced trials when compared with either the control or the displaced-control trials (Fig. 6 middle row: Watson–Williams test, *P* < 0.001). There was no significant effect due to species or a species and treatment interaction (*P* > 0.5). In addition, the heading angles for the two crayfish species in the displaced treatments were not significantly different from each other (*P* > 0.5).

### Walking speed

There was no change in the walking speed of the crayfish during the return path under any of the treatments (*P* > 0.6: Table 1), but *C. fodiens* had an overall faster walking speed than *F. rusticus* across all treatments.

## Discussion

The results of this study demonstrate that homing success rate, in both species of crayfish, is significantly diminished when a displacement occurs (Fig. 4). The possible mechanisms for homing for these crayfish can be categorized based on the type of cues they utilize, i.e., allothetic or idiothetic. Moreover, the manipulation in this experiment was designed to determine whether path integration could be a possible mechanism for homing for both primary and tertiary burrowers. This experiment found that displacing the crayfish during their outbound pathway created an error in the home vector and/or distance. This displacement significantly lowered homing success, created an induced error matching displacement, and increased heading angles relative to the home burrow. Given that our experimental manipulation altered only idiothetic information, the mistakes that crayfish made during the displaced treatment indicate that they are primarily using idiothetic information for homing purposes. These findings are consistent with the conclusion that both species of crayfish are using path integration to home and is further supported by previous work which demonstrated that these crayfish were *not* using cues directly associated with the burrow (Kamran et al. 2018). Our results indicate that *F. rusticus* were more successful in locating burrows than *C. fodiens* and thus, the homing ability of *C. fodiens* was more impaired than that of *F. rusticus*. Additionally, *C. fodiens* had a significantly larger induced error than *F. rusticus* and the induced error matched the displacement distance (Fig. 5). While displacement of the treadmill negatively impacted homing in both species, it is the primary burrowers that were more severely impacted by this linear displacement. Thus, the differences in homing ability between species may be related to their classification as primary (*C. fodiens*) or tertiary (*F. rusticus*) burrowers. Additionally, the reduced induced error in *F. rusticus* indicates possible compensatory mechanisms for translational error through a second homing mechanism.

In our study, the tertiary burrower (*F. rusticus*) displayed a reduced induction error. This reduced induction error indicates that *F. rusticus* may be possibly compensating for translational errors. As many animals employ secondary homing strategies to compensate for homing errors (Kim et al. 2010; Cheng et al. 2014; Murakami et al. 2017), it is possible *F. rusticus* also compensate through use of secondary homing strategies. Thus, when presented with conflicting sensory cues from the idiothetic information of a burrow location and the allothetic information about the burrow’s location, *F. rusticus* rely on allothetic information. This is consistent with conflicting cue studies in the desert ant, *Cataglyphis fortis*, which demonstrated that although path integration was the primary mode of homing, reliance on celestial compass took precedence over idiothetic cues (Lebhardt et al. 2012). Additionally, in cases where there is conflicting information from path integration and visual landmarks, desert ants were found to follow the landmarks (Collett et al. 1998). Evidence from work in coral reef fish demonstrated that fish rely on the number of sources of information such as odors from preferred food sources, conspecifics as well as visual cues when orienting, with responses to each of the stimuli being context dependent (Igulu et al. 2013).

As a result of these possible compensatory homing strategies, the primary burrowers were more negatively impacted than tertiary burrowers by the displacement. These findings are indicative of path integration as being a primary mechanism for short range homing in crayfish. Given that *C. fodiens* are a primary burrowing species and construct elaborate burrows they are likely to exhibit burrow fidelity, presumably due to the cost associated with the construction and maintenance of a burrow. *Faxoniusrusticus* on the other hand are tertiary burrowers which are more likely to occupy burrows constructed by others and thus display less of a fidelity toward specific shelters. Therefore, crayfish may use homing strategies that are a direct result of the time and energy investment in the construction of the burrow. Similar behaviors are found across species. Digger wasp’s ability to localize and defend a nest is tied to the investment in constructing the nest (Hoedjes et al. 2011). Species differences in spatial learning were found to be a factor in spatial cognition, with mound-building mice learning mazes significantly faster than non-burrow dwelling species (Bruck et al. 2017).

Primary burrowers use their burrows more frequently and leave on smaller foraging trips (Reynolds et al. 2013). As such, path integration may be a sufficient strategy to return to burrows without the accumulation of significant errors. Invertebrates such as fiddler crabs, crayfish, and spiders have been examined for their homing abilities on short range excursions, with evidence pointing to a home vector that is continually calculated and updated as the animals move (Seyfarth and Barth 1972; Layne et al. 2003a, 2003b; Seyfarth et al. 1982; Kamran and Moore 2015). In contrast, tertiary burrowers make longer foraging trips and previous research has demonstrated that when more distance is covered idiothetic cues are not effective and more robust allothetic cues are required (Cheung et al. 2007). Thus, tertiary burrowers may use information beyond idiothetic cues to perform their homing (Basil and Sandeman 2000; Tierney and Lee 2011; Tierney and Andrews 2013).

Within the landscape of these crayfish, numerous cues exist that may facilitate homing. For example, the burrows constructed by primary burrowers, and subsequently used by tertiary burrowers, often have structures such as “chimneys” associated with them (Berrill and Chenoweth 1982). These chimneys may provide a visual landmark cue for homing to tertiary burrowers. These dome-like structures have been observed in other invertebrate species such as fiddler crabs, where these domes appeared to play a role in courtship behavior (Kim and Christy 2015). Comparative research in two species of desert ants showed that these ants can alter homing strategies based on changes in their environment (cluttered versus featureless) (Bühlmann et al. 2011). Mantis shrimp can learn about sensory cues their surrounding and use this information to locate burrows (Reaka 1980). Thus, it is possible that these two species of crayfish have adopted two homing strategies based on their behavioral ecology of burrowing.

Other factors, beyond burrow construction and use, could play a role in the homing differences found within this study. While little is known about the biology and ecology of the crayfish, *C. fodiens*, these crayfish are less aggressive than *F. rusticus* (Guiasu et al. 2005). Differences in crayfish personality (e.g., bold/shy) that might exist across species could lead to differences in behavioral syndromes (Edwards et al. 2018). Underlying behavioral differences that manifest themselves in different tasks (social behavior, anti-predator, exploration) could alter the responsiveness and mechanisms of homing seen in this study (Sih et al. 2004; Sih and Bell 2008).

This study was designed to complement previous burrow displacement studies (Kamran and Moore 2015; Kamran et al. 2018). The results of this study demonstrate that there are differences in homing success rate, induced error, and heading angles in the two species of crayfish tested here. The difference in homing success rates was found to be consistent with prior studies (Kamran and Moore 2015), with the primary burrowing species *C. fodiens* appearing to rely on path integration, whereas the tertiary burrowing species, *F. rusticus*, relies on path integration as well as an unknown compensatory strategy. It is possible that the differences in homing strategies are related to their burrow use and energy investment in that burrow. Further investigation is needed to determine the secondary strategy that tertiary burrowers may be relying on.
